# Current status of skin cancers with a focus on immunology and immunotherapy

**DOI:** 10.1186/s12935-023-03012-7

**Published:** 2023-08-21

**Authors:** Mahsa Khayyati Kohnehshahri, Aila Sarkesh, Leila Mohamed Khosroshahi, Zanyar HajiEsmailPoor, Ali Aghebati-Maleki, Mehdi Yousefi, Leili Aghebati-Maleki

**Affiliations:** 1 Department of Microbiology, Faculty of Veterinary Medicine, Urmia University, Urmia, Iran; 2 Department of Cellular and Molecular Biotechnology, Institute of Biotechnology, Urmia University, Urmia, Iran; 3 Immunology Research Center, Tabriz University of Medical Science, Tabriz, Iran; 4 Student’s Research Committee, Tabriz University of Medical Sciences, Tabriz, Iran; 5 Department of Immunology, School of Medicine, Tehran University of Medical Sciences, Tehran, Iran; 6 Stem Cell Research Center, Tabriz University of Medical Science, Tabriz, Iran; 7 Department of Immunology, School of Medicine, Tabriz University of Medical Sciences, Tabriz, Iran

**Keywords:** Skin cancer, Melanoma, Non-melanoma skin cancer, Monoclonal antibody

## Abstract

Skin cancer is one of the most widespread cancers, with a significant global health effect. UV-induced DNA damage in skin cells triggers them to grow and proliferate out of control, resulting in cancer development. Two common types of skin cancer include melanoma skin cancer (MSC) and non-melanoma skin cancer (NMSC). Melanoma is the most lethal form of skin cancer, and NMSC includes basal cell carcinoma (BCC), squamous cell carcinoma (SCC), and other forms. The incidence of skin cancer is increasing in part owing to a demographic shift toward an aging population, which is more prone to NMSC, imposing a considerable financial strain on public health services. The introduction of immunostimulatory approaches for cancer cell eradication has led to significant improvements in skin cancer treatment. Over the last three decades, monoclonal antibodies have been used as powerful human therapeutics besides scientific tools, and along with the development of monoclonal antibody production and design procedures from chimeric to humanized and then fully human monoclonal antibodies more than 6 monoclonal antibodies have been approved by the food and drug administration (FDA) and have been successful in skin cancer treatment. In this review, we will discuss the epidemiology, immunology, and therapeutic approaches of different types of skin cancer,

## Introduction

**Fig. 1:**
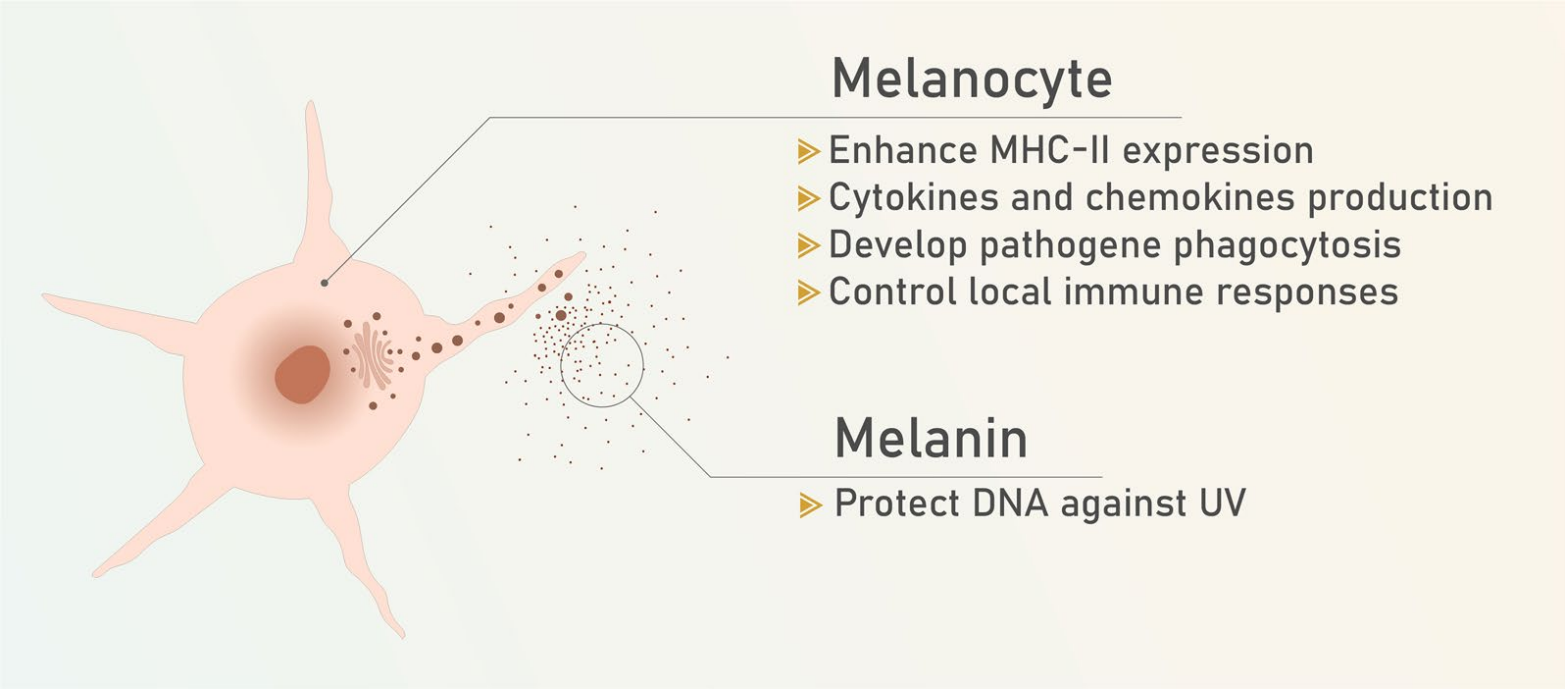
Melanocyte’s function in skin immunity. Melanocytes produce melanin which protects DNA from UV radiation and melanocytes enhance expression of MHC-II and produce cytokines and chemokines, develop pathogen phagocytosis and control innate and adaptive local immune responses

**Fig. 2:**
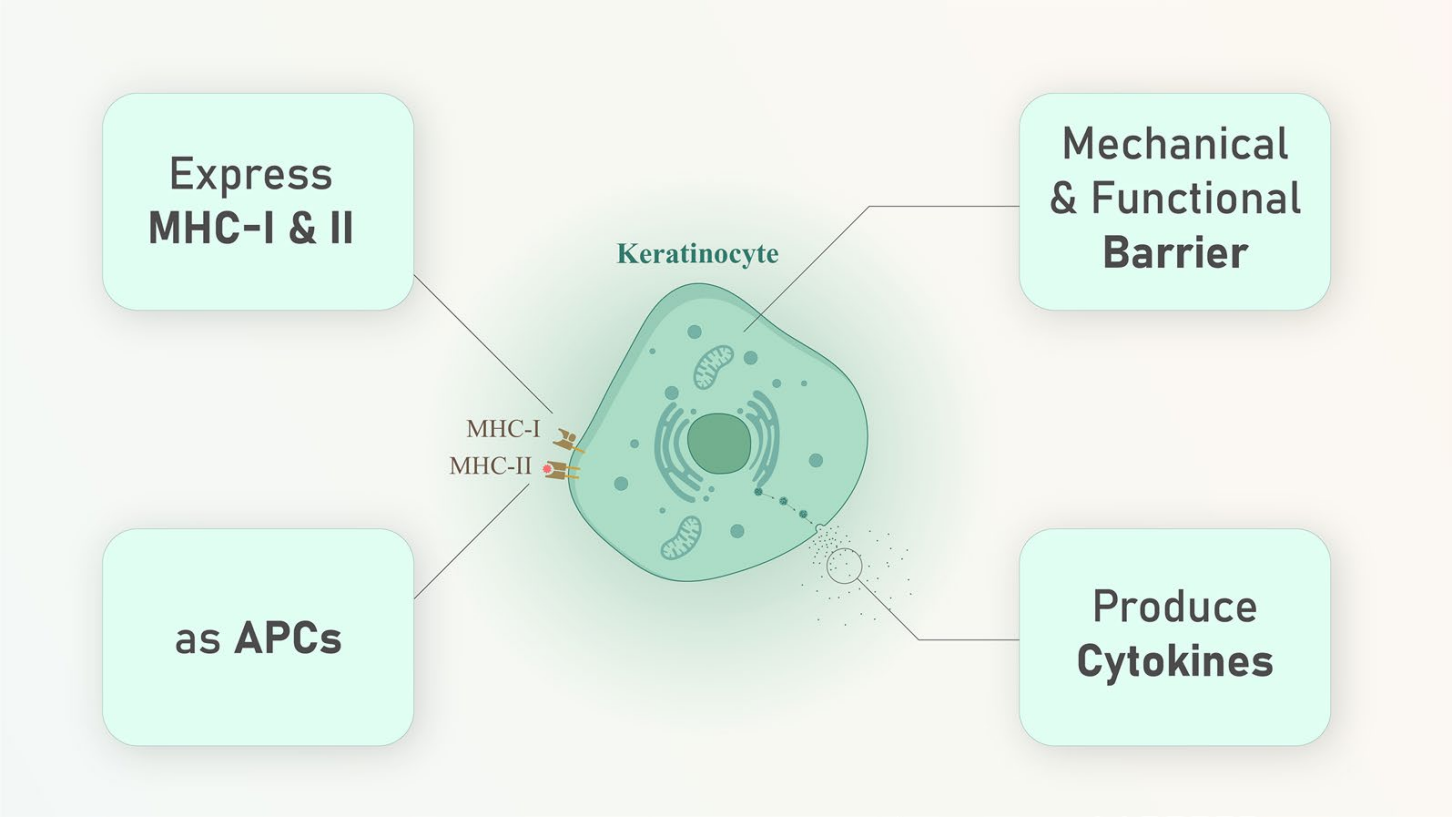
Keratinocyte’s function in skin immunity. Keratinocytes are important in maintaining the mechanical and functional barrier of epidermis and produce cytokines besides express MHC I and II and act as APCs and induce T cell responses by producing the cytokines

The skin is an organ that separates the body from the environment and serves as a barrier, protecting the body from UV waves, toxins, and infection [1]. The epidermis is the skin’s outer layer, which comprises various cells such as keratinocytes, melanocytes (Fig. 1), dendritic cells, Merkel cells, and Langerhans cells. The next layer underneath the epidermis is the dermis which consists of connective tissue, dermal dendritic cells, mast cells, and memory T cells [2, 3]. Skin cancer results when the DNA of the skin's cells got damaged and these damaged cells start to grow and divide without control and create a tumor (Fig. 2) [4]. Tumors could occur in various layers of skin and the most common layer is epidermis [5]. Skin cancer incidence is developing day by day. The main cause of skin cancer is UV exposure and because of ozone layer depletion, high rate of UV reaches to the surface of earth [6, 7]. Skin cancer usually is two types: (1) malignant melanoma and (2) non-malignant melanoma which is two types: BCC and SCC. BCC and SCC are in result of chronic UV exposure. The causes of malignant melanoma incidence are intense UV exposure and sunburn background [8, 9]. 80–85% of non-melanoma skin cancer are BCC and SCC. SCC is more dangerous and has high mortality rate [10]. Although there is discouraging increase in skin cancer incidence, the significant success of immunotherapies highlights the field. As an example, administration of decarbonize (DTIC) chemotherapy prior to ipilimumab with the combined checkpoint inhibitor immunotherapies ipilimumab and nivolumab has improved the 3 year overall survival for metastatic more over discovery of the CTLA4 (cytotoxic T-lymphocyte-associated antigen 4) and PD-1 (programmed-death 1) immune checkpoints in 2018 shed light to this field. Nivolumab alone or in combination with ipilimumab resulted in significantly longer progression-free survival than ipilimumab alone in patients with previously untreated metastatic melanoma. In patients with PD-L1-negative tumors, the combination of PD-1 and her CTLA-4 blocker was more effective than either agent alone [11].

## Skin cancer etiology

Skin cancer is a prevalent form of cancer among fair-skinned populations globally. The incidence and mortality rates associated with skin cancers are rapidly increasing, posing a significant threat to public health. As a result, healthcare systems aim to comprehend the underlying causes and development of skin cancer. Identifying causative factors is a crucial step towards preventing skin cancer. Excessive exposure to UV radiation accounts for almost 90% of all skin cancer cases [12]. Physiologically UV light plays a key role in vitamin D synthesis which is considered as an essential component of immune regulation in various conditions including infectious diseases and cancers [13]. However, excessive exposure of UV light is the main cause of skin cancer and both UV- A and UV- B play important role in skin cancer incidence [14]. Sunlight can be categorized into three groups based on wavelength: UVC (200–280 nm), UVB (280–320 nm), and UVA (> 320 nm). While UVC is filtered by the ozone layer and does not reach the Earth’s surface, a small percentage of UVA (1–10%) and the majority of UVB (90–99%) penetrate the Earth’s surface [12]. At first level, the UVB is the main cause of DNA damage and this damage could cause all phases of skin cancers (progression, promotion, and initiation) [15]. Because of the depletion of the ozone layer as a result of environmental changes, a high range of UV reaches to earth's surface and this is the reason for the increased incidence rate of skin cancer. Worldwide, studies suggested that there is a positive relation between skin cancer incidence and UV exposure history [16, 17]. UVB induces MAPK signaling pathway. It was shown that UVB is regulated by MAPK cascade and it includes ERK, JNK, and p38. In this simplified model the UVB activates acidic sphingomyelinase which activates ceramide and that is followed by activation of PKC. PKC phosphorylates and activates TCF and then connects to SRF in the site of SRE which is the promoter of a special gene. Activation of this pathway causes the expression of c-fos and in following that AP-1 gets activated. UVB also activates PKS and then JNK and JUN. Another effect of UVB is the activation of P38. UVB also could signal with Ras pathway. In the end, the result of the activation of this cascade is induction of cell responses. These responses include proliferation, differentiation, apoptosis, or tumorigenesis [7]. When a foreign antigen enters the human body, the immune system becomes activated and fights against that antigen but in case of UV light, this pathway does not occur because UV could suppress the immune system and it causes insufficient prevention against tumor development as well [18]. A subgroup of T regulatory cells is also induced to grow, specifically for the antigen encountered after UVR. In general, UV irradiation increases the number of T regulatory cells in the skin while decreasing the number of effector T cells, tipping the balance from T-cell-mediated immunity to immunosuppression [19]. This was seen in older individuals because they have weak immune systems. Immunosuppressive medicines such as cyclosporine, steroid, and azathioprine could defect the protection capacity of the immune system and as a result the incidence of various types of skin cancer increases [20, 21]. Skin cancer can also result from a mutation in the P53 gene. The P53 gene is crucial for maintaining genomic integrity, and it halts DNA replication in response to damage caused by various factors, including UV radiation [22, 23]. Normal exposure to ultraviolet (UV) radiation triggers a rise in P53 protein levels, which leads to the temporary arrest of cell division during the G1 phase. UV radiation-caused mutations in P53 are often linked to the carcinogenic effects of UV radiation and are believed to play a critical role in the development of squamous cell carcinoma (SCC). When exposed to UV radiation, our skin may also undergo photoaging, where oxidized lipid and metabolite aggregate levels increase, leading to autophagy loss and other cellular dysfunctions. While genetic predisposition isn’t always present or uniform across all non-melanoma skin cancers (NMSCs), SCCs can result from genetically predisposed clonal cell growth. Basal cell carcinoma (BCC) pathogenesis is caused by several tumor suppressor genes and proto-oncogenes, including members of the RAS family, Sonic Hedgehog pathway (PTCH1 and SMO), and the TP53 tumor suppressor gene. Impaired activation of the Sonic Hedgehog pathway appears to be a crucial component in BCC carcinogenesis. However, SCCs are driven by several mutated genes. Furthermore, certain oncogenic viruses such as HPV, EBV, and the recently discovered Merkel Cell Polyomavirus (McPhee) have been found to possess oncogenic potential for NMSCs. The E6 and E7 oncoproteins produced by HPV can integrate into the host's keratinocyte genome. Notably, HPV-positive NMSC typically exhibits a more benign clinical behavior than HPV-negative NMSC [24, 25].

## Types of skin cancer

Skin cancer includes both malignant melanoma (MM) and nonmelanoma skin cancer (NMSC) which shows the most malignancy in Caucasians [26, 27]. The incidence of both MM and NMSC is developing. The incidence of MM among adults ˃50 years old showed 3.6 times increase annually [28]. Non-melanoma skin cancer (NMSC) is a prevalent type of cancer that is frequently diagnosed. Basal cell carcinoma (BCC) and squamous cell carcinoma (SCC) are the most common types of NMSCs, accounting for 70% and 25% of cases, respectively, although skin cancer can originate from any skin cell in the host. Both BCC and SCC have favorable prognoses, particularly when identified early, despite exhibiting different behavior, growth patterns, and metastatic potential. It's worth noting that BCC contributes minimally to NMSC mortality rates [29]. The incidence of metastatic basal cell carcinoma (BCC) is very rare, occurring in only 1 out of 14,000,000 cases, while 2 out of 14,000,000 patients with locally advanced BCC will die from it. In contrast, squamous cell carcinoma (SCC) has a variable metastatic rate ranging from 0.1 to 9.9%, accounting for approximately 75% of NMSC-related deaths. Surgical resection remains the primary treatment method, but several alternative approaches have been reported for treating NMSCs, such as photodynamic therapy, cryotherapy, topical imiquimod 5%, and topical diclofenac sodium 3% [30].

### Basal cell carcinoma (BCC)

Most common type of skin cancer is BCC which is highly developed in human population [12]. Basal cell carcinoma (BCC) is responsible for 80% of all skin cancers. It is a unique type of skin cancer that originates in the basal cells found in the lowest layer of the epidermis. BCCs typically appear as red-colored spots on the face and scalp, and their development period is slow. While BCCs have a low potential for spreading to other areas, they can invade neighboring tissues and spread to different parts of the body if left untreated [31]. BCC often occurs on head and neck [9, 32]. BCC can be classified into three distinct types: superficial, nodular, and sclerosing/morphea type. Superficial BCC appears as erythematous plaque on the tip of the nose and extremities. Nodular BCC typically appears as pearlescent papules or nodules with rolled margins on the head and neck. Morphea type BCC is characterized by scar formation, making it difficult to diagnose through observation alone. Individuals with Gorlin syndrome are frequently associated with BCC, and these patients often exhibit BCC lesions on their face or other anatomical areas. [33]. Non melanoma skin cancer such as BCC and SCC originate from keratinocytes [34, 35]. Prognostic factors about BCC are tumor size, histologic subtype, tumor site, margins and recurrence [14]. Currently, two therapies targeting the hedgehog pathway are FDA-approved for the upfront treatment of recurrent, metastatic, or locally advanced BCC not possible to surgery or radiation. The hedgehog signaling pathway is often dysregulated in BCCs through mutations in either PTCH1 or SMO genes. Vismodegib was the first hedgehog inhibitor (HHI) approved by the FDA in 2012 based on the phase II ERIVANCE (NCT00833417) trial (Table 1). An ORR of 47.6% for locally advanced BCC and 30% for metastatic BCC was observed at 12 months [24, 25]. After a follow-up period of 39 months, the updated trial results indicated an overall response rate (ORR) of 60.3% and 48.5% for locally advanced and metastatic BCC, respectively. Sonidegib is the second FDA-approved HHI designed for oral administration and is used as the first-line treatment for BCC (according to Table 1). The drug was approved in 2015 for treating locally advanced BCC that has recurred after surgery or radiation therapy, or for patients who are not eligible for either treatment approach. During the phase II BOLT (NCT01327053) pivotal trial, sonidegib achieved an ORR of 56.1%, with a median duration of response of 26.1 months, and a 93.2% 2 year survival rate for locally advanced BCC. However, the ORR for metastatic BCC was only 7.7% [36].

**Table 1:** Approved monoclonal antibodies for skin cancer treatment by FDA

Approved mABs	Mechanism	Type of SC	Other forms
Cemiplimab-rwlc	Anti PD-1	Basal Cell Carcinoma Cutaneous Squamous Cell Carcinoma	Libtayo
Pembrolizumab	Anti PD-1	Cutaneous Squamous Cell Carcinoma Melanoma Merkel Cell Carcinoma	Keytruda
Ipilimumab	Blocks CTLA-4	Melanoma	Yervoy
Nivolumab	Anti PD-1	Melanoma	Opdivo
Avelumab	Anti PD-1	Metastatic Merkel cell carcinoma	–
Cetuximab (chimeric)	Anti PD-1	Advanced non melanoma skin cancer	–

### Squamous cell carcinoma (SCC)

Incidence of this type is fewer than BCC. It develops between squamous cells and in dry and rough areas [12]. However, it could develop in some parts of the skin which have more sunlight exposure. It could appear in form of red spots. Similar to BCC, it could spread to other parts of the body but some treatments were discovered to prevent the development [37]. BCC converting to metastatic form is rare however it seems to have more mortality rate but SCC metastases rapidly and the possible cause of this high rate is chronic sunlight exposure, it also could be seen in every part of the body which is in exposure to sunlight [34, 35]. In blacks and Asian Indians, SCC accounts for 30–65% of diagnosed skin cancer cases. Certain factors increase the risk of developing SCC, such as Fitzpatrick skin types I and II, outdoor work, human papillomavirus (HPV) types 16, 18, and 31, hereditary conditions like albinism, xerodermatic pigments, and epidermal dysplasia verruciform. Some genetically inherited skin disorders also increase the risk of SCC. However, prolonged exposure to UV radiation from the sun is the most significant risk factor. A direct correlation has been observed between psoralen and UVA (PUVA) exposure and SCC incidence. SCC typically develops in sun-exposed areas, with approximately 55% of all SCCs affecting the head and neck region. Additionally, SCCs often occur on the extensor surfaces of the hands and forearms (18%), while up to 13% of SCC cases occur on the legs [38, 39].

### Melanoma

It is a type of skin cancer that appears in melanocytes of the skin [12]. Although that is rare but it has higher mortality rate in compare to other types because it could spread with lymphoid capillaries [37, 40]. It occurs in every age group but older individuals are affected more [41]. If it was diagnosed in the early stages it will follow up with effective treatment. Signs and symptoms of melanoma includes changes in size, shape and color of mole, presence of leak or bleeding site in mole, the mole is itchy, hard, and lumpy [14, 30]. Melanoma is a less common form of skin cancer than other types, but it can be more dangerous if not detected early and has a high mortality rate of 75%. This type of skin cancer is associated with melanocytes in the epidermal layer, which synthesize melanin pigments to protect the skin from mutagenic UV radiation. Compared to basal cell carcinoma (BCC) and squamous cell carcinoma (SCC), malignant melanoma is less commonly diagnosed. There is currently no clear treatment procedure for melanoma, so prevention is the best approach for controlling this disease. [38, 41, 42]. Other abnormal types of skin cancers have been recognized but they are really rare, for example, lymphoma [31].

### Epithelial skin cancer and oncogenic virus infection

Non-melanoma skin cancers, such as BCC, SCC, and AK, are prevalent among transplant patients. It has been found that AK is a precursor to both BCC and SCC, and its incidence among transplant patients occurs 15 years earlier than in individuals without transplants. AKs have a higher prevalence among heart transplant recipients than those with kidney or spleen transplants. In contrast to normal individuals, SCC is more commonly diagnosed in transplant patients than BCC [43, 44].

### Kaposi sarcoma

This type is usually seen in older individuals. It is often because of a virus named KS which is associated with the human herpesvirus (KSHV) and it was named HHV- 8. The lower limb, trunk and extremity of the upper limb are the purpose of Kaposi sarcoma, albeit, it could include mouse, lymph nodes, and stomach [45].

### Neuroendocrine skin cancer (Merkel cell carcinoma)

Usually, it is seen on the head and neck of older individuals. Immune system suppression plays an important role in incidence of this type of skin cancer. It is reported that MCC in transplanted patients is more than in normal individuals [46].

## Epidemiology

Skin cancer includes both malignant melanoma (MM) and nonmelanoma skin cancer (NMSC) which shows the most malignancy in Caucasians [26, 27]. The incidence of both melanoma (MM) and non-melanoma skin cancer (NMSC) is on the rise. Among adults aged over 50, there has been an annual increase of 3.6 times in the incidence of MM. In 2016, the estimated number of new cases of skin cancer was 76,380, accounting for 45% of all new cancer cases. It's important to note that the reported incidence of melanoma may be underestimated because the National Cancer Registries have identified a lack of accurate statistical data from some countries [28].

### Melanoma

The increasing rate of melanoma incidence is not companion with mortality rate which is because of increased diagnosis potential or more biopsies [28].

### Non-melanoma skin cancer

NMSC includes Bowen’s disease, BCC, and SCC. The incidence of NMSC is higher in Caucasians than in MM, and although the epidemiology of NMSC due to geographic differences is cautious, the lower mortality rate of NMSC has led to a higher incidence of NMSC and a higher statistical record. Become. Than others [47–49]. NMSC is the most expansive cancer in Australia. In the USA it was estimated that the annually cost of NMSC is 650 milion dolor, which means the treatment cost of NMSC is 6–7 times more than the treatment cost of melanoma [50–52].

### Causes of increased prevalence of skin cancer

Prevalence increasing about skin cancer associated with multiple factors such as transfer of human population to an older population who is more talented in the incidence of NMSC [53]. However, studies indicated that increased therapeutic and occasional usage of UV light plays an important role [54]. Skin cancer is the most frequently diagnosed malignant neoplasm among the white population. The prevalence rates of melanoma and non-melanoma skin cancers are on the rise globally. Therefore, it is essential to study and comprehend the current epidemiological status of skin cancer to facilitate effective management of the disease [55]. Melanoma is more frequently diagnosed among individuals of the white population compared to other races. The risk of developing melanoma increases with age, with an average age at diagnosis being around 60 years. Men have a higher incidence rate of melanoma compared to women, with a 1.5 times greater frequency of diagnosis. Although there is no difference in incidence rates for men and women until the age of 40, after the age of 75, the incidence rate of melanoma in men is 3 times higher than in women. [56, 57]. Moreover, incidence frequency is related to skin color and geographical area [49]. Increased incidence along with high prevalence causes more costs which were forced a significant economic burden on public health services. The measurable economic burden includes direct costs as a result of medical care and indirect costs associated with a decreased potential capacity of life duration and production [50].

### NMSC risk factors

The development of an effective skin cancer prevention strategy requires a comprehensive understanding of the risk factors involved. Non-melanoma skin cancers (NMSCs) can be categorized into two groups: individual and environmental factors. The most notable individual risk factors include age, gender, and genetics. Meanwhile, exposure to ultraviolet (UV) radiation is the primary environmental factor. The incidence of squamous cell carcinoma (SCC) increases at a faster rate with age than basal cell carcinoma (BCC). While NMSC incidence is relatively equal between genders during adolescence, after 45 years of age, keratinocyte carcinoma development in men is 2–3 times higher than women. Moreover, genetic predisposition is primarily linked to the number of melanocytes in the skin or an individual's ability to tan [50].

## Skin immune system

**Fig. 3:**
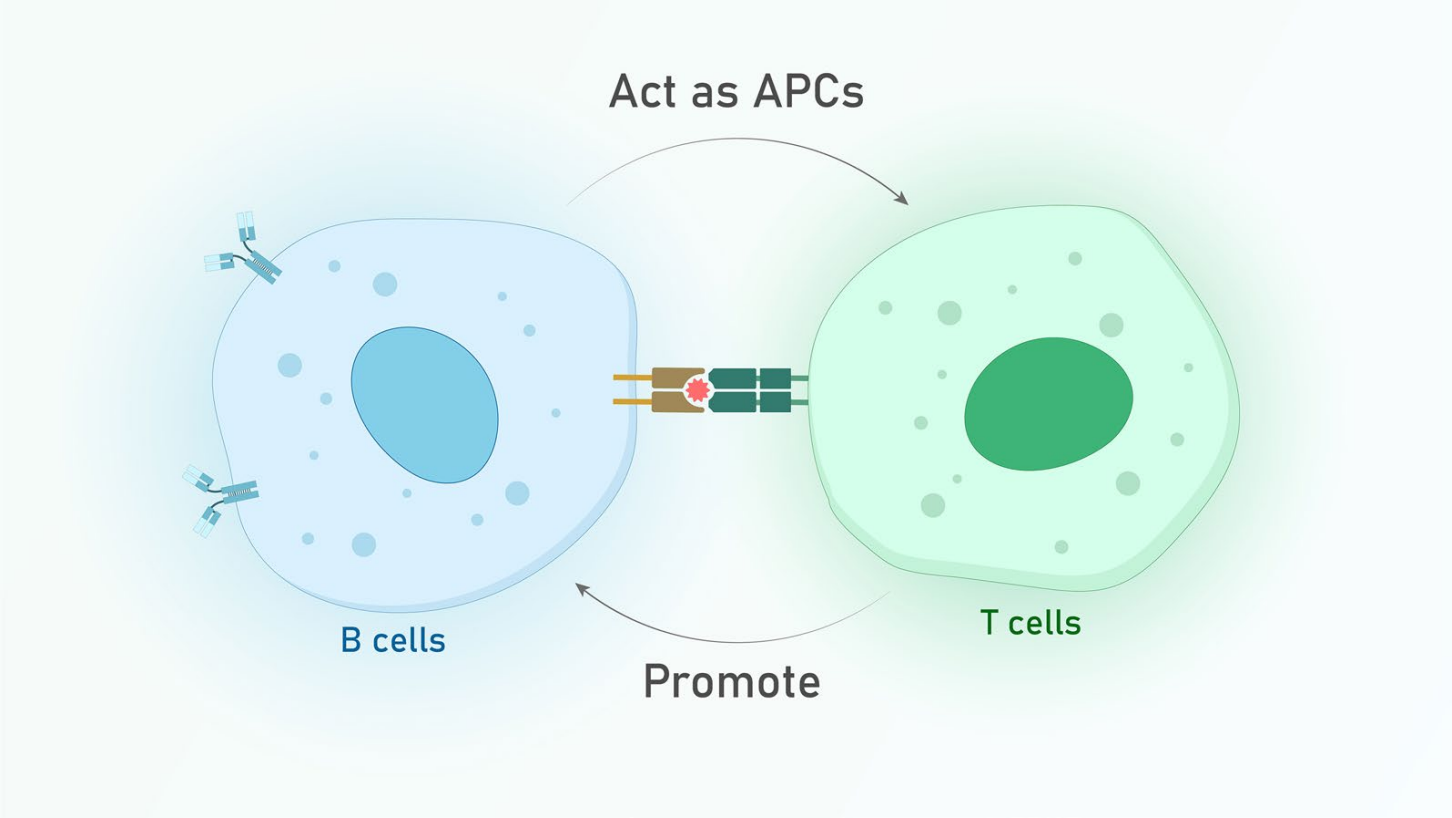
Relation between B and T cells in skin. B cells can also play the role of APCs for naive T cells and activated CD4^+^ T cells promote naive B cells to be activated

The development of an effective skin cancer prevention strategy requires a comprehensive understanding of the risk factors involved. Non-melanoma skin cancers (NMSCs) can be categorized into two groups: individual and environmental factors. The most notable individual risk factors include age, gender, and genetics. Meanwhile, exposure to ultraviolet (UV) radiation is the primary environmental factor. Squamous cell carcinoma (SCC) and basal cell carcinoma (BCC) are the two most common types of non-melanoma skin cancer (NMSC). While both SCC and BCC are more likely to occur as people age, SCC tends to increase at a faster rate with age than BCC. Additionally, men have a higher risk of developing keratinocyte carcinomas, including both SCC and BCC, after the age of 45 compared to women. This gender difference in incidence is believed to be due to differences in sun exposure, hormonal factors, and genetic predisposition. Genetic factors do play a role in NMSC development, but the exact mechanisms are not yet fully understood. Some studies have suggested that the number of melanocytes in the skin or an individual’s ability to tan may be linked to genetic predisposition for NMSC [58]. The epidermis consists of different types of cells such as keratinocytes, melanocytes, and Langerhans cells. Melanocytes are responsible for producing melanin that protects the skin from the harmful effects of UV radiation. Additionally, they aid in stimulating the expression of MHC-II proteins, generating cytokines like IL-1β, IL-6, TNF-α, and chemokines. With these functions, melanocytes help to develop pathogen phagocytosis and regulate innate and adaptive local immune responses. [59, 60]. The role of keratinocytes in maintaining the mechanical and functional barrier of the epidermis is crucial, as they not only express MHC I and II but also produce cytokines. This makes them key players in the pathophysiology of infectious and inflammatory processes. Under normal conditions, keratinocytes secrete IL-1α and IL-1β in an inactive state. However, when exposed to UV radiation, inflammasomes are activated, leading to the release of IL-1β. Additionally, keratinocytes can function as antigen-presenting cells (APCs) and induce CD4 + and CD8 + T cell responses by producing Th1 and Th2 cytokines [61, 62]. Also, there are T lymphocytes in basal and corneum layers and most of them are CD8 + lymphocytes. Langerhans cells act as a population of both DCs and macrophages and they disperse in the epidermis. Merkel cells along with LCs are responsible for skin sensibility (Fig. 3). There are various types of immune cells population in the dermis which are specialized immune cells such as DCs, CD4 + T lymphocytes, γδ T lymphocytes, natural killer cells (NK), macrophages, and mast cells [63–67]. Innate and adaptive immunity is activated in an effort to limit cancer pathogenesis [67]. Innate immunity triggers adaptive immunity for elimination the cancer via more specific immune mechanisms [68, 69]. Genetic and epigenetic make some modifications in cancer cells [70]. These modifications are associated with changes in the composition of cell surface proteins which cause the expression of tumor-associated antigens and complement proteins can recognize these antigens [71]. Complement cascade also promotes some of the effects of adaptive immune responses since this cascade can activate B and T cells and heighten their survival rate. CRP- mediated complement may also cause expansion of B and T cells which could target cancer cells [72]. Cancer cells alter or reduce the expression of MHC I which causes activation of NK cells by NKG2D on the surface of NK [73]. These cells induce apoptosis by various mechanisms such as cytoplasmic granules which are dependent on TNF-α release, antibody dependent complement cytotoxicity, and cytokines such as IFN-γ. IFN-γ mediates activation and maturation of DCs or other APCs [73]. Neutrophils as another tool of innate immunity contribute to cancer progression. Proteases of neutrophils facilitate growth and metastasis of cancer cells [74]. Some other cells cause evolutionary links between innate and adaptive immunity. For example, dendritic cells and macrophages act as APCs and activate adaptive immunity [75, 76]. Tumor cells upregulate expression of OX40L on DCs and other APCs which causes costimulatory signals for T cells. These costimulatory signals make T cells differentiate into Th2 T cells [77]. NKT cells as innate immune cells activate NK cells or CD8^+^ T cells by IFN-γ secretion and they mediate tumor lysis by granzymes or perforin [78]. Interaction between NKT and DC by CD40 signaling enables activation and secretion of IL-12. CD40 ligand expresses on NK cells, mast cells, macrophages, B cells, epithelial cells, endothelial cells and activated T cells which bind to CD40 on APCs and cause costimulatory signals [76, 77]. In addition, NK and CD8^+^ T cells could be activated by IL-12 and as a result that tumor lysis and cancer progression suppresses [78]. γδ T cells similar to NK and CD8^+^ T cells interact with MICA/B on tumor cells by expressing NKG2D and causes secretion of perforin proteins and subsequently tumor lysis [79]. They also secrete IFN-γ which activates NK or CD8^+^ T cells and γδ T cells mediate antibody dependent complement cytotoxicity by CD16 which recognizes tumor associated antigens [79]. Adaptive immunity has a critical role in tumor eradication or proliferation depending on the environmental signals [69, 80]. Adaptive immunity uses antibodies, T and B cells, and APCs to produce sufficient responses against specific antigens of cancer cells [81]. These neoantigens are formed due to tumorigenesis/oncogenesis process which is phagocytosed by APCs or pinocytosis by DCs and presented to B or T cells [68].

B cells can also play the role of APCs for naive T cells and activated CD4^+^ T cells can also promote naive B cells to be activated [79]. Another way of B cell activation is T- independent way. In this way, antibodies are secreted and bind to tumor derived antigens. Following this binding, ADCC or CDC could be initiated and lysis tumor cells [82]. or NK cells could be activated via Fc receptors [83]. Antigen-specific T cell receptor recognizes MHC class I/tumor antigen complex CD8^+^ T cells got activated and induces catalytic CD8^+^ T cell- mediated lysis of cancer cells [68]. There is an important topic about innate immunity which is about immune checkpoints that mediate either cancer progression or regression [77, 84]. The provision of appropriate costimulatory signals is essential for the activation of naive T cells. In their absence, T cells may become anergic or develop immune tolerance to cancer cell-associated antigens. [81]. Similarly, the CTLA4 which binds to the CD80/86 proteins on APCs may causes tolerance in T cells [77, 84]. Cancer cells express CTLA4 too and this mechanism corresponds with immune tolerance and causes cancer progression [85]. Another receptor found on T cells is PD-1, which binds to PD-L1 on APCs and plays a role in mediating immunosuppression. PD-1 is not only expressed by T cells but also by other immune cells such as B cells, NK cells, monocytes, dendritic cells, and Tregs [84]. Researchers indicated that PD-1 is expressed by cancer cells and they escape immunity by this mechanism [84, 85]. Cancer cells also secrete CCL22 which recruits Tregs to suppress the function of immune system especially T cells [86, 87].

## Immunotherapy for advanced skin cancer

Immunotherapy is a critical treatment for various types of cancer, including skin malignancies such as melanoma, SCC, BCC, and MCC. The European Society of Skin Oncology guidelines suggest that anti-PD-1 immunotherapy is the primary treatment for patients with locally advanced cSCC (la-cSCC) who are not suitable candidates for radical surgery or radiotherapy, as well as those with metastatic cSCC (m-cSCC). Ultimately, effective treatment will depend on individual patient factors and should be determined in consultation with a healthcare professional [88] Selective treatment is essential when tackling medical conditions. However, sometimes systemic therapeutic regimens such as EGFR inhibitors and platinum-based chemotherapy (especially cisplatin) are utilized, but their effectiveness may be limited [89] Currently, cemiplimab is the only anti-PD-1 agent approved for treating both locally advanced and metastatic cSCC in the USA and Europe. Its use is being evaluated in adjuvant and neo-adjuvant therapy as well. For locally advanced and metastatic BCC (la-BCC and m-BCC), Sonic Hedgehog inhibitors (HHIs) are the mainstay of systemic therapy. However, there is increasing evidence supporting the potential role of anti-PD-1 regimens, particularly in tumors resistant to HHIs or in patients who cannot tolerate treatment due to adverse effects. One of the most significant challenges since the introduction of immunotherapy is identifying the ideal candidates who would benefit from this type of treatment [90]. It’s important to note that immunotherapy can have severe, irreversible, or even fatal side effects. As a result, it’s crucial to conduct a thorough risk–benefit evaluation before starting treatment. In most cases, immune checkpoint inhibitors (ICIs) are used when dealing with high disease burdens in metastatic or locally advanced settings. However, the use of ICIs as adjuvant or neoadjuvant regimens for NMSCs is not currently approved. When selecting patients for immunotherapy, the primary goal is to maximize the benefits while minimizing adverse effects. Identifying biomarkers or other parameters that can predict therapeutic efficacy or the likelihood of therapy failure or adverse effects is critical. Unfortunately, there are limited clinical predictors available to define immunotherapy responses. It's well-known that ICIs can cause autoimmune-like toxicities, which are referred to as immune-related adverse events. Therefore, close monitoring is necessary during and after treatment, and timely intervention can be crucial in managing such adverse events. By carefully considering the risks and benefits, healthcare providers can make informed decisions regarding the use of immunotherapy for their patients [91]. The adverse effects of immunotherapy vary depending on the agent, type of cancer being treated, and individual susceptibilities. They can affect any organ in the body, although cutaneous and intestinal manifestations are the most prevalent. While corticosteroids are useful for treating moderate and severe immune-related adverse events (irAEs), the occurrence of these events may sometimes necessitate discontinuing immunotherapy. Additionally, irAEs are not useful predictors because side effects do not emerge until after treatment has begun [92].

### Immune checkpoint inhibitors

ICI represents a major advance in cancer treatment According to new data, it has been confirmed that treatment with anti-PD-1 monotherapy can be effective in managing metastatic BCC, KS, and cutaneous angiosarcoma. Though there is still a lack of well-established research on the subject, combining anti-CTLA-4 and anti-PD-1 checks has been shown to have the best 5 year overall survival rate among other ICIs therapies [93]. This therapy has also proven successful in treating advanced melanoma, including melanoma brain metastasis. Recent studies have revealed that around 40% of patients with metastatic melanoma who underwent PD-1 inhibitor monotherapy experienced positive outcomes, while over 60% of those who underwent standard therapy combined with dual blockade of CTLA-4 and PD-1 showed favorable results [94]. In March 2017, Avelumab, an anti-PD-L1 agent, received accelerated FDA approval for the treatment of metastatic MCC. The Merkel 200 trial's JAVELIN (NCT02155647) Part A is an open-label, single-arm study that focused on patients with chemotherapy-resistant metastatic MCC (Table 1). The ORR reported was 33% (23.3–43.8%) with a CR of 11.4%, and Grade 3–4 AEs were reported at 10.1%. Although ICI has been successful in eliminating cancer by boosting immune responses to tumor-associated antigens, the toxicity of ICI treatment is outweighed only by its efficacy. ICI treatment may induce autoimmune toxicity because of its mechanism of action. These immune-mediated adverse events can affect any organ system (most commonly skin, colon, endocrine system, and liver) and appear to mimic classic autoimmune disorders. irAEs may occur in a significant proportion of patients (10–90% in recent studies), and the spectrum of systemic irAEs differs for each ICI agent. Pembrolizumab toxicities are most commonly reported as arthritis, hepatitis, and pneumonia, whereas nivolumab mainly causes endocrine disturbances. Ipilimumab induces cutaneous, gastrointestinal, and nephrotoxicity. Reports show different toxicity situations based on the paradigm of ICI use, such as a single agent, in combination with other ICIs, or in combination with different agent classes. Adverse event severity appears to increase significantly with protocol combination. Experience with ICI treatment in oncology has shown that toxicity from ICIs usually affects multiple organs and occurs primarily within the first few months of treatment. IrAEs caused by anti-CTLA-4 agents occur earlier and dose-dependently, while those caused by anti-PD-1 and PD-L1 agents occur later and dose-independently [95]

### Monoclonal antibodies

Monoclonal antibodies play different roles in cancer prevention [96]. Monoclonal antibodies are a type of targeted therapy used to treat cancer. They are designed to recognize and attach to specific proteins on the surface of cancer cells that can trigger an immune response or directly inhibit the growth of cancer cells. Monoclonal antibodies help the immune system in several ways, such as by marking cancer cells with a “flag” to make them easier for immune cells to identify and attack. This process is known as antibody-dependent cell-mediated cytotoxicity (ADCC). Furthermore, they can be engineered to carry toxins or radioactive particles, which can deliver targeted radiation or chemotherapy directly to cancer cells.Monoclonal antibodies have shown promise in treating a range of cancers, including leukemia, lymphoma, breast cancer, and colorectal cancer. They offer various benefits over traditional chemotherapy, such as fewer side effects and more precise targeting of cancer cells. Additionally, researchers are continually developing new monoclonal antibodies that target different proteins involved in cancer growth and progression, providing hope for improved treatment options in the future [97]. Ipilimumab is the first line of developed immunotherapy factors which causes increasing in the viability rate of cancer patients. In comparison to anti-CTLA-4, Pembrolizumab, Nivolumab, and anti-PD-1 antibodies which are associated with increased viability of patient with metastatic melanoma, the Ipilimumab is on first stage of wild melanoma treatment with metastatic BRAF. Combination usage of these medicines along to anti-CTLA-4 antibodies boosts the immune responses but they also increase toxicity [98]. Avelumab is the monoclonal antibody that blocks PD-1 and was approved by FDA in case of metastatic Merkel cell carcinoma treatment [99].

Treatment of nonstop basal cell carcinoma is limited and now there is not any recommendation for the patients who do not respond to hedgehog inhibitors (saridegib, sonidegib, vismodegib). Expression of PD-L1 was reported in neoplastic cells and intra-tumor lymphocytes in BCC [98]. Moreover, complete clinical responses to Pembrolizumab were described in patients with metastatic BCC. There is not any public agreement about management of metastatic or nonstop SCC [98]. A recent study showed increased expression of PD-1 and its ligand in 38 biopsy samples of 24 patients with SCC whom most of them were at high risk. PD-1 expression in tumors with perineural invasion was dominant [100]. Monoclonal antibodies are a type of targeted therapy used to treat cancer. They are designed to recognize and attach to specific proteins on the surface of cancer cells, which can trigger an immune response or directly inhibit the growth of the cancer cells. Monoclonal antibodies help the immune system in several ways, such as by marking cancer cells with a “flag” to make them easier for immune cells to identify and attack, a process known as antibody-dependent cell-mediated cytotoxicity (ADCC). Additionally, they can be engineered to carry toxins or radioactive particles that deliver targeted radiation or chemotherapy directly to cancer cells. Monoclonal antibodies have shown promise in treating a range of cancers, including leukemia and lymphoma, breast and colorectal cancer [99].

Immune checkpoint blocking by monoclonal antibodies appeared as a successful treatment for patient with melanoma which targets cytotoxic T lymphocyte antigen (CTLA-4) directly, for instance, Ipilimumab that targets PD-1 and PDL-1. Ipilimumab was the first inhibitor of CTLA-4 which enhanced viability and responses against tumors in patients with developed melanoma [101]. In September 2014, FDA approved Pembrolizumab as the first inhibitor of the PD-1 for patients with unresectable and metastatic melanoma treatment. In the case of an increased number of patients with metastatic melanoma who showed durable responses by immunotherapy, studies discovered synergism between immune checkpoint inhibitors which target CTLA-4, PD-1, and PDL-1 [102].

In December of 2014, the FDA granted approval for Nivolumab, commercially known as Opdivo, for the treatment of metastatic melanoma in patients who had previously been treated with Ipilimumab and also for those who had become BRAF V600 mutation positive after treatment with a BRAF inhibitor. Nivolumab is a monoclonal antibody that functions by blocking PD-1, which can lead to a reduction in tumor growth rate based on its effectiveness in syngeneic mouse tumor models. In March of 2015, the FDA granted approval for Nivolumab’s use in treating lung cancer as well [103]. In September 2018, FDA approved Cemiplimab-rwlc (libtayo) for developed cutaneous SCC (CSCC) treatment. Cemiplimab is an intravenous humanized monoclonal antibody that targets PD-1 receptor. Cemiplimab blocks T cells inactivation process and improves anti-tumor responses of immune system. Checkpoint inhibitors present worthwhile improvements in cancer treatment [104].

**Fig. 4:**
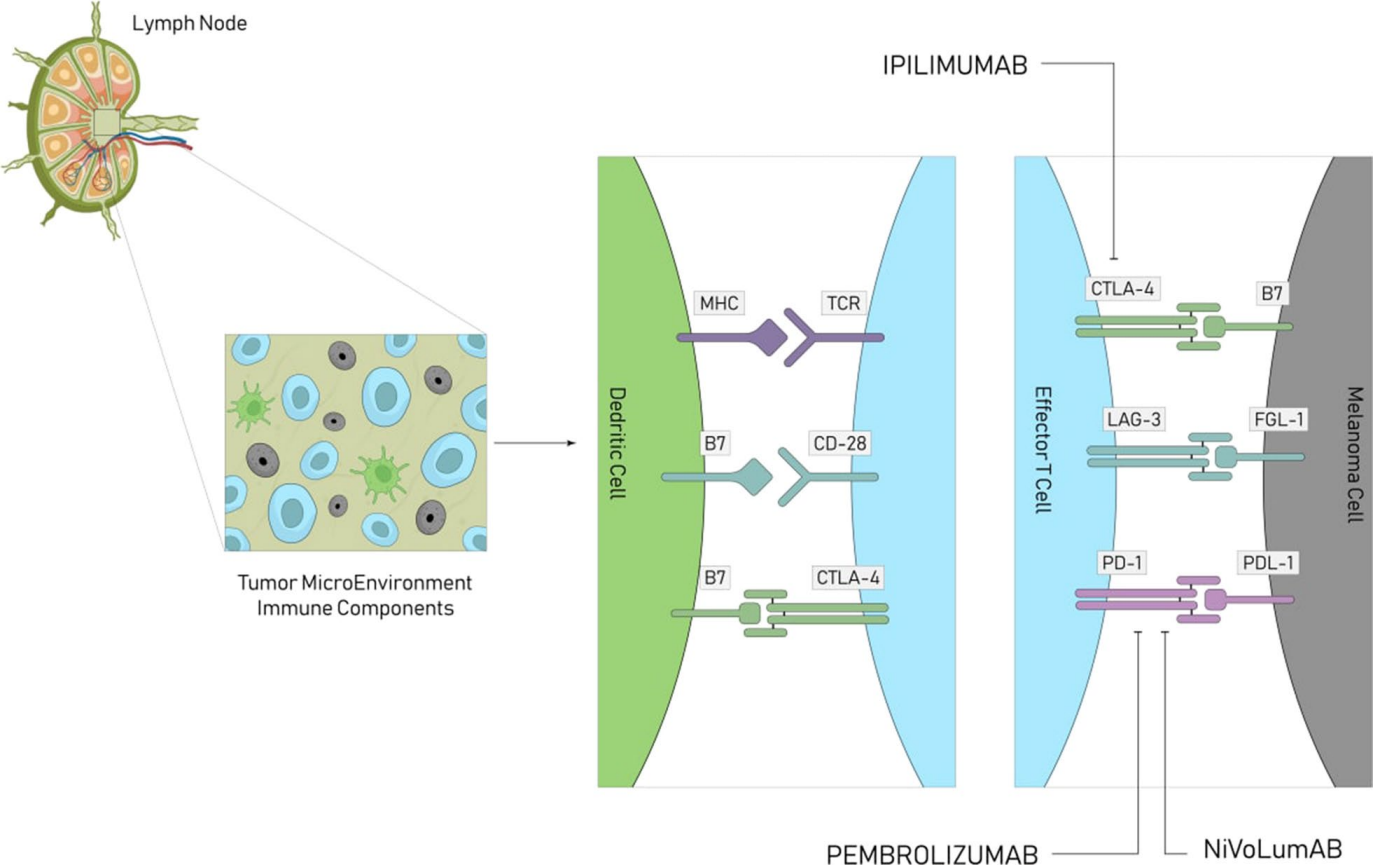
Immune check point inhibitors: Recent evidence confirmed the significant clinical benefit from using immune check point inhibitors,this figure demonstrates the mechanism underlying its beneficial effect

**Fig. 5:**
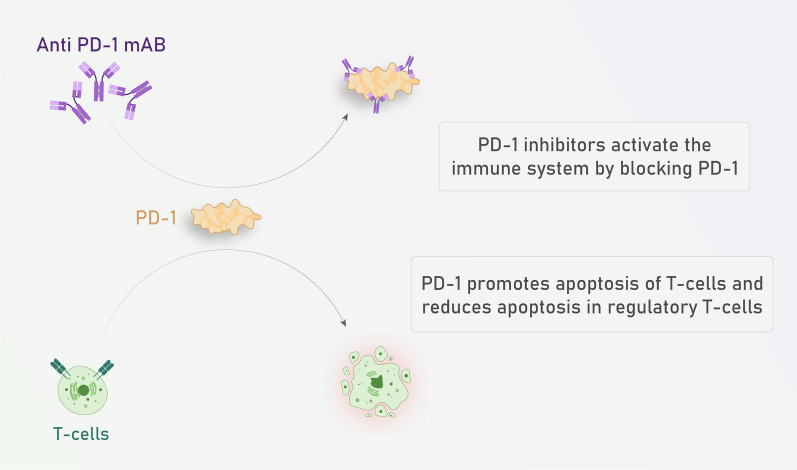
Ant- PD-1 mechanism of action.Programmed cell death protein 1 (PD-1) is an immune checkpoint and causes apoptosis of antigen-specific T-cells in lymph nodes and reduces apoptosis in regulatory T cells. PD-1 inhibitors block PD-1 and activate the immune system to attack tumors

Chimeric monoclonal antibody Cetuximab targets epidermal growth factor receptor in advanced non-melanoma skin cancer. Most major risk factors about skin cancer include: more than 50 years old, I or II phenotype of Fitzpatrick, increased natural or artificial UV exposure, immune system suppression, solid tissue grafts, and tanning beds [105]. Cetuximab is a chimeric monoclonal antibody against epidermal growth factor receptor and FDA approved it for treatment of head and neck SCC associated with radiotherapy [106] (Figs. 4, 5). There are several factors than can determine the efficacy of immune check point inhibitors. TILs (CD4, CD8), growth factors, Tregs, and checkpoints (PD-1, CTLA-4), are linked with the efficacy of checkpoint inhibitors in SCC and thus are effective prognostic markers. Among the TILs, CD3, CD8 (effector/cytotoxic), and CD4 (helper) T cells are found to have an anti-tumor immune response and are correlated with SCC patients’ favorable outcomes [107]. In line with this finding, it is found that TAMs derived from melanoma have the potential to express PD-1 which leads to M2 polarization. Thus administration of an anti-PD1 antibody can repolarize and activate TAMs to release sCD163 in melanoma patients [108].

### T cell therapy

Targeting specific antigens using T-cell infusions has demonstrated potential in the treatment of HIV and cancer. This approach, combined with immune checkpoint blockade, is leading a shift in the paradigm of cancer immunotherapy. The use of genetically modified T cells that express chimeric antigen receptor (CAR) genes is considered the most promising strategy among these methods. CARs consist of a single extracellular chain, and T cells are transfected with CAR constructs using plasmid transfection, mRNA, or viral vectors. Since its inception, the CAR structure has undergone significant changes. In this composition, only the CD3 signaling domain is involved, which is recognized as the “first-generation CAR.” [109]. Since then, in order to improve T-cell proliferation and persistence, costimulatory end domains Second- (e.g., CD3 plus 41BB- or CD3 plus 41BB-) or third- (e.g., CD3 plus 41BB- or third-generation (e.g., CD3 plus CD28-signaling domains) CARs with signaling domains 41BB and CD28) CARs have also been used in the past [109]. Although regional lymph node involvement (stage III) is a part of the metastasis process, only stage IV, in which tumor cells metastasize to distant organs, is considered metastatic melanoma according to the staging, the lymphatic system is the main pathway by which melanoma cells move, despite the hematogenous system occasionally being implicated [110]. CMV antigen has been detected in several malignancies including gliomas and melanoma, which has prompted researchers to target these antigens as a therapeutic approach [111, 112]. In this way recent studies have discovered an effective approach MCMV-derived CD8 + T-cell peptide epitopes as cytotoxic and immunomodulatory agents to promote immediate tumor control and long-term anti-tumor immunity conducive to melanoma cancer [112].

### Cytokines

Cytokines can have opposing effects—either defending the body against diseases or promoting inflammatory processes that exacerbate sickness. In recent decades, pure recombinant cytokine mediators have been developed and explored as a treatment for various malignancies, including melanoma. Immunotherapy using cytokines is a promising approach to melanoma treatment as it has the potential to activate host immune cells that specifically target malignant cells while preserving normal cells.

IFN-γ, for example, may decrease the growth of melanoma cells and angiogenesis while enhancing apoptosis by directly affecting cells. However, data from various studies reveal that IFN-γ’s immunomodulatory functions are critical in mediating its anticancer effects. The ability of IFN-γ to stimulate NK cell-mediated cytotoxicity and proliferation is crucial to its anticancer activity. This cytokine also promotes the production, activation, and proliferation of memory CD8 + cytotoxic T lymphocytes (CTLs). Additionally, IFN-γ has been shown to enhance the immunogenicity of tumor cells by altering the expression of surface molecules. Previous research has demonstrated that the therapeutic response to IL-2 immunotherapy is mediated by the proliferation and activation of cytotoxic cells within host tissues [113]. Although the exact signaling pathways that mediate these effects remain unknown. Finally, the fact that IL-2 therapy can elicit such potent antitumor action in a subset of patients goes against what is known about the cytokine's cellular targets. For example, IL-2 can boost the survival and proliferation of CD4 + CD25 + Foxp3 + Tregs, which suppress T-cell function and antitumor immune responses by suppressing T-cell function. Modest-dose IL-2 produces low response rates and appears to be ineffective in metastatic melanoma while being less toxic and more convenient. Clinical trials are presently being conducted to test newer, more novel treatments such as IL-2 gene therapy and strategies to reduce the toxicity of this medication [114]. Monocytes, macrophages, dendritic cells, and other antigen-presenting cells are involved in the synthesis of IL-12. IL-12’s potent immunomodulatory properties lead to increased T and NK cell proliferation in response to it. One of IL-12’s most important features is its ability to cause immune effector cells to produce IFN-γ, which mediates cytotoxic immune responses. It also increases the expression of a number of extracellular molecules involved in antigen presentation, such as MHC class I and II molecules, as well as cellular motility, including ICAM-1. Importantly, IL-12 has been found to promote the production of anti-angiogenic chemokines such as IP 10 and MIG in an IFN-dependent manner.

IL-21, on the other hand, has a broad range of pharmacological effects on various cell types and has been shown to be anticancer in preclinical melanoma models. It triggers mortality in naive B lymphocytes and those activated via Toll-like receptor (TLR) 4 or TLR9 ligands, while it stimulates proliferation and antibody isotype switching in reactivated B lymphocytes via CD40 ligands. The proliferative effects of IL-21 on CD40-activated B cells are consistent with its ability to promote the formation of mature antibody-producing plasma cells [100]. A Phase I trial in Europe in which patients with metastatic melanoma received subcutaneous IL-21 was conducted to investigate an alternative way of delivery and the MTD of subcutaneous delivery was determined to be roughly 200 g/kg. After 8 or 16 weeks of treatment, one melanoma patient had a partial response, while the other six had stable disease. Several more Phase II multicenter trials assessing intravenous delivery of the cytokine to patients with metastatic melanoma are currently underway or have just concluded. Other studies on the impact of IL-21 in ongoing trials have recently been reported at ASCO, with moderate results in patients [101].

### Cancer vaccines

Cancer vaccines are developed to activate tumor-specific CD8 + CTL. The most common immunization techniques use MHC class I restricted peptide epitopes of TAA. To enhance presentation by endogenous APCs in vivo, these vaccines have been administered with various adjuvant formulations that include cytokines and Toll-like receptor (TLR) ligands. Peptide-based vaccination benefits from the current data on MHC class I peptide binding motifs of the most common HLA types. Algorithms can screen protein amino acid sequences for TAA-derived peptide epitopes, and GM-CSF is one such approach. Clinical trials using syngeneic, autologous, or allogeneic tumor cells transfected to express high levels of GM-CSF have resulted in successful immunological and clinical responses [115]. Utilizing autologous tumor antigens by harnessing a personalized approach appears to be a promising strategy for cancer treatment. There are two examples of such strategies, including ex vivo loading of APCs with tumor lysates and fusion of tumor cells and autologous APCs. In rare cases, immunity to unknown tumor lysates and foreign helper proteins has been observed. Additionally, APCs (autologous or derived from allogeneic cell lines) can be loaded with tumor genomic DNA using autologous tumor cells. This enables the processing and presentation of uncharacterized mutant gene products that are specific to the tumor for immune activation.Moreover, the use of pathogens in cancer vaccines can significantly enhance immune responses when tumor antigens are presented. One promising vaccination modality is mRNA vaccines due to their inherent immunogenic features. mRNA is a non-infectious, non-integrating platform that is degraded by normal biological processes [116]. It is also an easily adaptable, stable, and highly translatable vaccination platform. Biotech research has demonstrated that intravenously delivered RNA-lipoplexes, known as RNA-LPX, may successfully target DCs in vivo. The LPX protects RNA from extracellular ribonucleases and ensures that it is efficiently taken up and expressed by APCs. Most importantly, they discovered that tumor antigens encoded by RNA-LPX elicit potent antigen-specific effector and memory T cell responses in three melanoma patientsSurvivin, a member of the Inhibitor of Apoptosis Protein (IAP) family, exhibit a dysregulated expression profile in several autoimmune diseases and malignancies including SCC [117, 118]. This feature makes survivin a target to develop survivin-targeting vaccines to treat malignancies such as melanoma, in a recent phase II study a vaccine using the peptides Sur1M2 and IDO5 was combined with the chemotherapy temozolomide (TMZ) for treatment of metastatic melanoma patients in order to target several immune inhibiting mechanisms and the highly malignant cells expressing survivin. Immunological analyzes showed vaccine-specific responses in 8 of the 12 patients tested (67%), a significant decrease in CD4 + T-cell frequencies during treatment, a trend toward decreased naive CD4 + and CD8 + T-cell frequencies, and showed an increase in preservation frequency CD4 + and CD8 + T cells [119].

### Oncolytic viruses

Engineered oncolytic viruses represent a new treatment option for patients with metastatic melanomas. Due to their diverse oncolytic and immunogenic properties, these drugs offer a promising add-on to existing systemic therapy while also providing a single-agent alternative for those who might not tolerate conventional therapies for advanced disease. Studies have demonstrated that T-value VECs are effective in treating melanoma, and current clinical trials exploring their use in combination with checkpoint inhibitors are expected to further enhance their effectiveness. When used alongside checkpoint blockade, oncolytic virus therapy appears to be a potential treatment option for both locally progressed and late-stage melanoma [120].

## Conclusion

Skin cancer is one of the most common cancers that causes great loss. UV exposure is the main reason skin cancer develops, so it is easily preventable, but the increasing incidence of skin cancer has necessitated multiple treatment options. Surgical treatment remains the main treatment modality, but new therapeutic innovations remain important to reduce morbidity and mortality. [121, 122]. Immune system in some cases makes a large number of antibodies that attack foreign substances. Antibodies circulate throughout the body until stick to the specific antigen and starts cascades that destroy the cells containing the antigen by immune system. Recently researchers could design antibodies targeting a certain antigen and monoclonal antibodies same as these antibodies are designed to identify the right antigen to attack. This ability was also used in cancers treatment. Monoclonal antibody sometimes targets the cancer cells specially and in other cases act like immunotherapy and trigger immune cells to respond and fight with cancer cells [123]. In some cancers, monoclonal antibodies are approved to use as treatment include; brain cancer, breast cancer, chronic lymphocytic leukemia, colorectal cancer, head and neck cancers, Hodgkin’s lymphoma, lung cancer, skin cancers, non-Hodgkin's lymphoma, prostate cancer, and stomach cancer. Over the last three decades, monoclonal antibodies have been used as powerful human therapeutics besides scientific tools. Along with the development of monoclonal antibody production and designs procedures from chimeric to humanized and then fully human monoclonal antibodies, more approved monoclonal antibodies are available in the market for the treatment of various diseases [123]. Monoclonal antibodies were also successful in treatment of some skin cancers and they were approved by FDA. Sometimes monoclonal antibodies cause allergic reactions or other side effects include fever, Chills, Weakness, Headache, Nausea, Vomiting, Diarrhea, and Low blood pressure but compared with chemotherapy drugs these side effects are fewer. This management makes one method brighter and more suitable for research and experimentation [122].

## Data Availability

Data sharing is not applicable to this article as no new data were created or analyzed in this study.
